# Effectiveness of Dupilumab in the treatment of both atopic dermatitis and alopecia universalis

**DOI:** 10.1002/ccr3.2915

**Published:** 2020-05-25

**Authors:** John E. Call, Sakshi Sahni, Kathryn A. Zug

**Affiliations:** ^1^ Department of Dermatology Dartmouth Hitchcock Medical Center Lebanon NH USA; ^2^ Department of Medicine University of Illinois at Chicago Chicago IL USA

## Abstract

Recent studies have demonstrated significant molecular parallels between atopic dermatitis and alopecia areata as predominantly TH2‐mediated diseases. This case report highlights the potential for Dupilumab as a targeted biologic therapy in both atopic dermatitis and alopecia areata.

## Graphical abstract

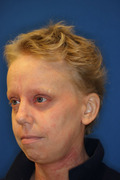

## 1 INTRODUCTION

Dupilumab is a novel human monoclonal antibody approved for treatment of moderate‐to‐severe atopic dermatitis (AD). Recent studies investigating the pathophysiology of alopecia areata (AA) have demonstrated significant molecular parallels to that of AD, raising the question of Dupilumab's therapeutic potential in nonscarring autoimmune alopecia. We report a patient who demonstrated dual efficacy of Dupilumab in both AD, as well as longstanding alopecia universalis (AU).

## 2 CASE REPORT

**FIGURE 1:**
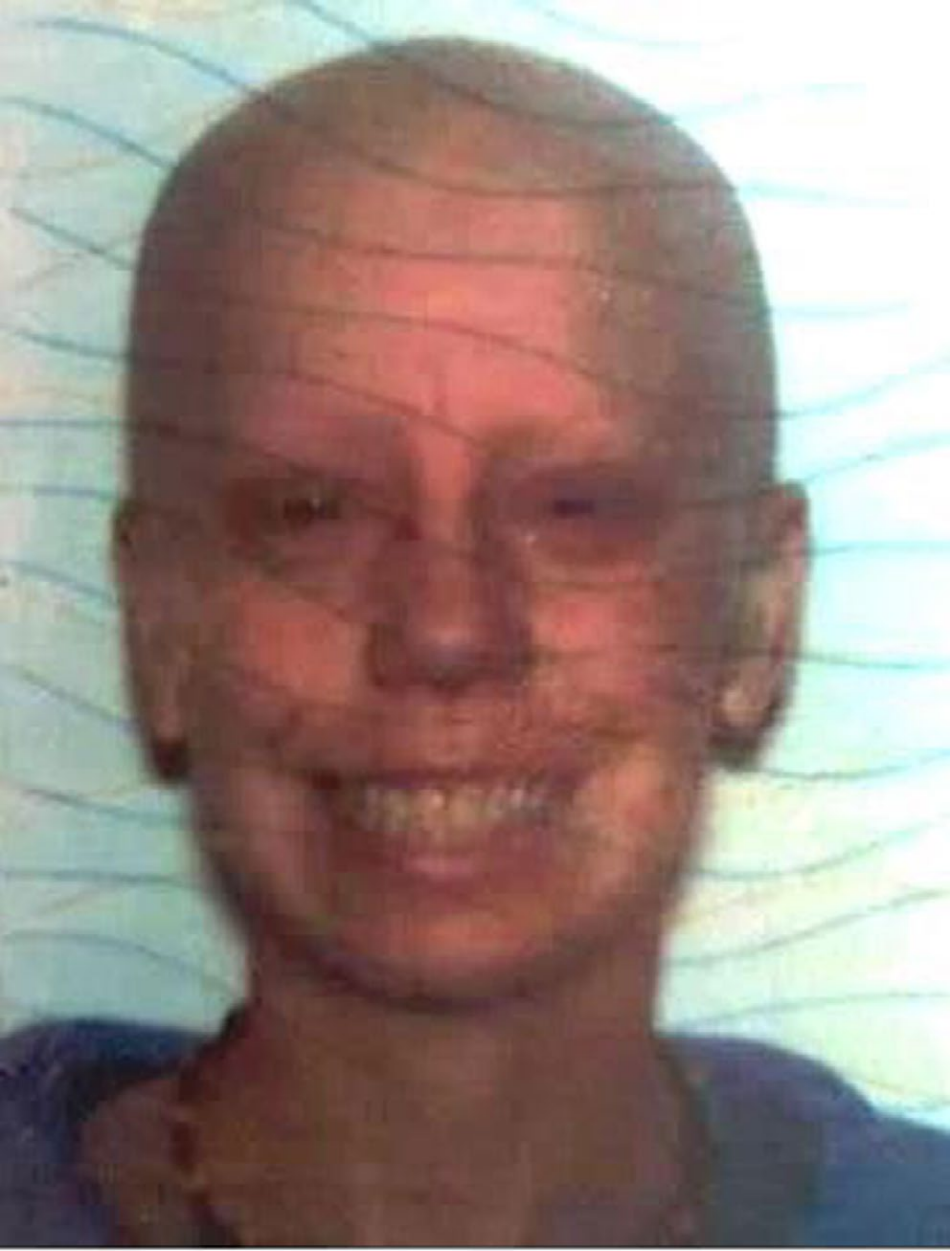
Passport photograph demonstrating alopecia of the scalp and brows approximately 12 mo prior to therapy with Dupilumab

A 34‐year‐old woman with past medical history of severe recalcitrant AD since early childhood and exercise‐induced asthma presented to clinic with a worsening flare of atopic dermatitis. The patient had previously used potent topical steroids, topical calcineurin inhibitors, and methotrexate with limited benefit. The patient also presented with a concomitant history of longstanding alopecia since the age of seven years old that began on the scalp and over 12 months progressed to AU (Figure 1). She described several intermittent periods of partial patchy regrowth on the scalp, yet no full remission. She had also taken prednisone as a teenager for AU which temporarily provided limited regrowth although relapsed once the corticosteroid was discontinued.

**FIGURE 2:**
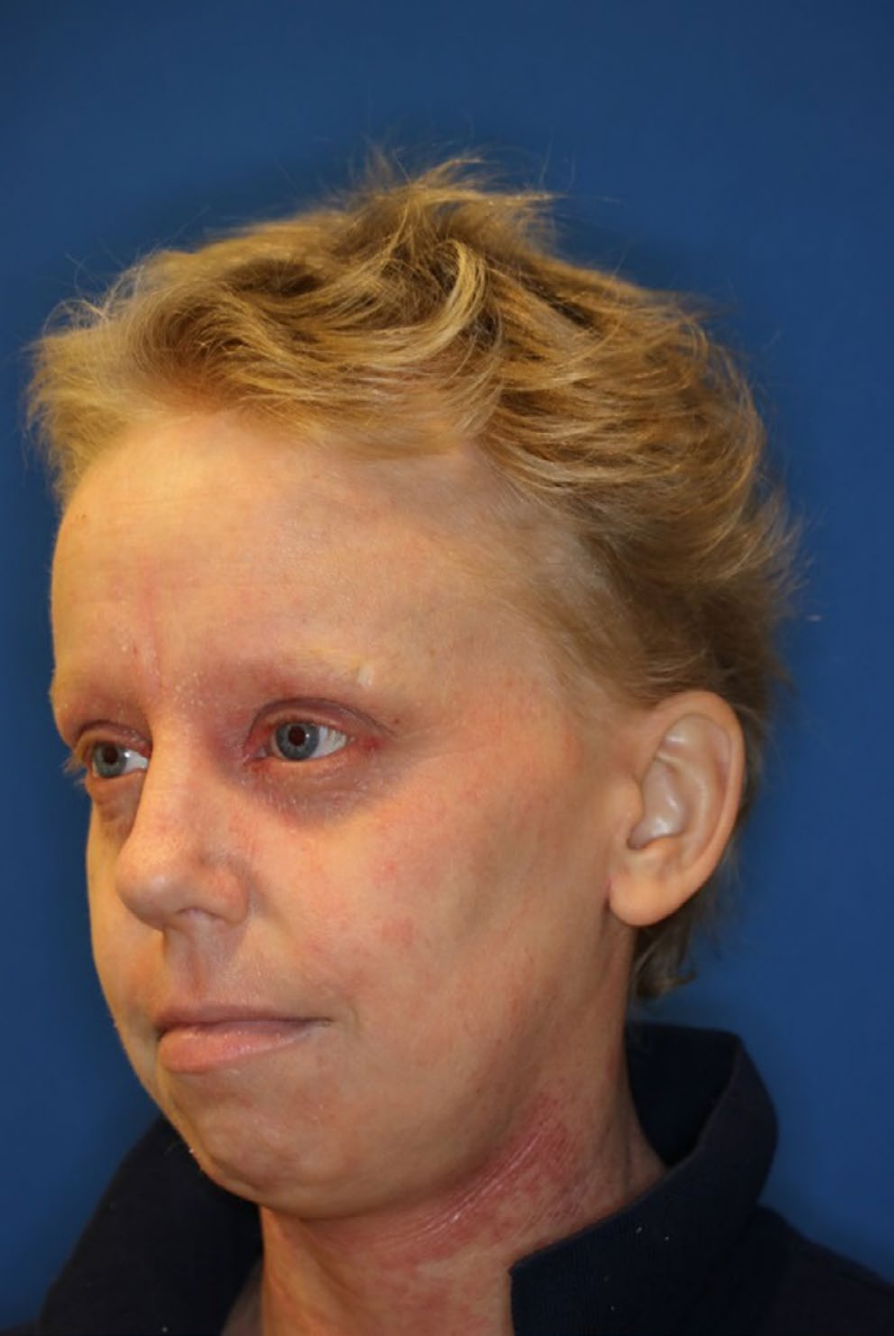
Patient with regrowth of terminal hair on scalp, eyelashes and brow after 10 mo of Dupilumab therapy

On the initial visit, she presented with diffuse eczematous papules and plaques most prominent on the back, abdomen, and bilateral upper extremities. She was also noted to have extensive nonscarring alopecia with complete hair loss of the scalp, eyelashes, brow, axillae, forearms, and legs. Dupilumab treatment was initiated with a loading dose of 600 mg subcutaneously followed by 300 mg once every other week. Within 6 weeks, the patient's AD significantly improved and she began to notice regrowth of sparse vellus hairs, initially on the scalp, followed by the eyebrows and eyelashes. Within ten months, the patient had regained a complete remission of her AU with total regrowth of course terminal hair on the scalp, face, forearms, pubic area, and legs with excellent control of her skin disease (Figure 2).

## 3 DISCUSSION

Dupilumab has affirmed key components in the TH2 immune response by targeting the α‐ subunit of the interleukin (IL)‐4 receptor, the driving force of Th2‐cell differentiation, leading to inhibition of IL‐4 and IL‐13 cytokine signaling. Developing theories have questioned whether this novel biologic could be utilized in other diseases. Recently, a strong association has been confirmed between AD and various autoimmune diseases, in particular AA.^1^ Studies have demonstrated higher risk of developing AA in patients with either personal or family history of an atopic diathesis. These patients also have the propensity for more severe alopecia (universalis or totalis) compared with baseline.^2^ Moreover, recent advances have been able to further characterize the immunologic composition of AA highlighting new understanding in the intersection between the pathophysiology of AA and AD, specifically the Th2 helper cell. Both IL‐4 polymorphisms and IL‐13 gene susceptibility have been recognized with AA.^1^ Other studies have demonstrated the IL‐13 cytokine to be significantly upregulated in both AD and AA lesions compared to nonlesional skin.^1^, ^3^ This was further reinforced by decreased expression of Th2‐related genes following steroid injections in AA patients.^4^ Finally, a unifying molecular etiology between the two diseases is further supported with the advent of novel JAK‐STAT pathway inhibitors for both AA and AD.^5^, ^6^ IL‐4 and IL‐13 proinflammatory cytokines have been shown to depend on the JAK‐STAT pathway to elicit their pathophysiologic functions.^7^ By inhibiting the interleukin 4 alpha receptor with Dupilumab, intracellular signaling via the JAK‐STAT pathway is diminished with improvement in both hair growth and atopic disease. With this knowledge, antagonism of these Th2 immunologic pathways has given rise to the theoretical therapeutics of Dupilumab in the setting of AA.

Succeeding Darrigade et al^8^ and Alniemi et al,^9^ this is one of the few reported cases to exhibit clinical efficacy of Dupilumab in reversing AU. We recognize that studies have, conversely, demonstrated Dupilumab inducing AA.^10^ However, this further emphasizes the need for further investigation, such as the ongoing NIH‐funded randomized, placebo‐controlled pilot study involving patients receiving Dupilumab for moderate‐to‐severe AD with concomitant AA.

## CONFLICT OF INTEREST

None declared.

## AUTHOR CONTRIBUTIONS

JEC and KAZ: had full access to all of the data in the study and took responsibility for the integrity of the data and the accuracy of the data analysis. JEC, SS and KAZ: involved in study concept and design; acquisition, analysis, and interpretation of data; drafted the manuscript; involved in critical revision of the manuscript for important intellectual content; made administrative, technical, or material support. KAZ: Supervised the study.
